# Poly(ADP-Ribose)Polymerase Activity Controls Plant Growth by Promoting Leaf Cell Number

**DOI:** 10.1371/journal.pone.0090322

**Published:** 2014-02-28

**Authors:** Philipp Schulz, Karel Jansseune, Thomas Degenkolbe, Michaël Méret, Hannes Claeys, Aleksandra Skirycz, Markus Teige, Lothar Willmitzer, Matthew A. Hannah

**Affiliations:** 1 Bayer CropScience NV, Innovation Center, Zwijnaarde, Belgium; 2 Department of Molecular Systems Biology (MOSYS), University of Vienna, Vienna, Austria; 3 Max-Planck-Institute of Molecular Plant Physiology, Potsdam-Golm, Germany; 4 Department of Plant Biotechnology and Bioinformatics, Ghent University, Ghent, Belgium; 5 Department of Plant Systems Biology, VIB, Ghent, Belgium; Universidade Federal de Vicosa, Brazil

## Abstract

A changing global environment, rising population and increasing demand for biofuels are challenging agriculture and creating a need for technologies to increase biomass production. Here we demonstrate that the inhibition of poly (ADP-ribose) polymerase activity is a promising technology to achieve this under non-stress conditions. Furthermore, we investigate the basis of this growth enhancement via leaf series and kinematic cell analysis as well as single leaf transcriptomics and plant metabolomics under non-stress conditions. These data indicate a regulatory function of PARP within cell growth and potentially development. PARP inhibition enhances growth of *Arabidopsis thaliana* by enhancing the cell number. Time course single leaf transcriptomics shows that PARP inhibition regulates a small subset of genes which are related to growth promotion, cell cycle and the control of metabolism. This is supported by metabolite analysis showing overall changes in primary and particularly secondary metabolism. Taken together the results indicate a versatile function of PARP beyond its previously reported roles in controlling plant stress tolerance and thus can be a useful target for enhancing biomass production.

## Introduction

The growth and biomass production of a plant is the result of balancing the genetic potential with environmental factors such as soil composition, pathogens, light and water availability. To maximize their fitness under variable conditions plants have evolved several regulatory systems to optimize their energy use and to tolerate or acclimate to diverse stresses. In order to stabilize crop yield and plant biomass for energy production in a changing global environment, the understanding of these systems and their regulation is of great interest for biotechnology [1]. This is a challenging topic since reproducible and stable effects on plant growth in agriculture are usually smaller [2] than in highly controlled lab conditions as seen for example in [3].

Extreme conditions such as drought stress have a strong impact on plant growth and development and these effects are mediated by interconnected signaling pathways involving hormones as well as metabolism, both of which regulate growth also under normal conditions [4]–[8]. The growth impact of these internal and external factors is mediated via the complex and modular cell cycle [9]–[11]. In the context of growth regulation poly-(ADP-ribose)-polymerases (PARPs) have usually only been recognized as a factor relating to stress tolerance. PARP proteins were first described in plants 15 years ago [12]–[15] although PARP activity was described earlier [16]; [17]. These proteins are characterized by the PARP signature [18] and are known for their ability to post-translational modify target proteins by adding ADP-ribose polymers (PAR). For this Nicotinamide adenine dinucleotide (NAD+) is used destructively, thereby linking PARP activity with cellular energy homeostasis and consequently cell death processes [14]; [5]. Overall the function of PARP and PAR in plants is poorly understood, particularly in contrast to human and animal science, and the need for further investigation has been recently highlighted [19]. In mammals, 18 PARP or PARP-like proteins are so far described [20] and are linked to several processes as recently reviewed like DNA damage repair, transcriptional regulation, chromatin status, the circadian clock, metabolism or the proteasome [21]–[24]. In Arabidopsis the three PARPs that are likely to have catalytic activity (PARP1-3) [25] are mainly assigned to tolerance of abiotic stress [5]; [26]–[29]. In addition PAR level and PARP were linked to biotic stress responses [30], [31] where a reduced callose deposition was observed upon flg22 and elf18 application when applied in combination with a chemical PARP inhibitor. Moreover, PARP's and PAR were associated with different developmental processes such as flowering and trachea element differentiation [32]; [33]. The various functions of PARP have been suggested to be mechanistically related to DNA repair [26]; [5]; [34], transcriptional regulation [35]; [32]; [36]; [27] interplay with abscisic acid (ABA) [27], energy homeostasis, cell death [14]; [37]; [5]; [38] and, more recently, redox homeostasis [39]; [29]. Collectively this demonstrates that PARP and PAR are deeply involved in plant homeostasis and response regulation and that the processes it effects, including hormone and energy homeostasis, redox balance, DNA repair and transcription control are all also important for cell and overall growth regulation.

The described involvement of PARP in plant response pathways and the initially observed growth enhancement prompted us to investigate the underlying physiology in more detail. In order to elucidate the mechanisms underlying the non-stress growth enhancement we established an assay system enabling a time-resolved phenotypic and molecular analysis. We used this system to perform a systematic evaluation of physiological and molecular responses to understand the observed growth enhancement of PARP inhibited plants.

## Materials and Methods

### Growth conditions

Arabidopsis (*Arabidopsis thaliana* – ecotype *Columbia* (*Col-0*)) were grown in sterile *in-vitro* conditions on ½ Murashige-Skoog [40] with 1.0% glucose or in hydroponics with mineral nutrient solution, in 80–100 µE light, at 22–23°C and a long day regime (16 h/8 h). As mineral nutrient solution a modified Hoogland's solution was used with final concentrations of KNO_3_ (1,5 mM), Ca(NO_3_)_2_ (0,75 mM), (NH4)_2_SO_4_ (0,5 mM), MgSO_4_ (0,75 mM), K_2_HPO_4_ (0,5 mM), FeEDTA (0,1 mM), H_3_BO_4_ (50 µM), MnSO_4_ (10 µM), ZnSO_4_ (2 µM), CuSO_4_ (1 µM), Na_2_MoO_4_ (0,1 µM) and Na_2_O_7_S (1 µM). The PARP inhibitor 3-Methoxybenzamide (3MB) was dissolved in 125 µl DMSO and added to the growth media to a final concentration of 0.2 mM. All chemicals were obtained from Sigma-Aldrich™. The short-term treatments where performed by initially growing the plants on nylon meshes with 1 µM pore size (SEFAR), on standard media in control conditions and transferring the mesh and seedlings [41] to the respective PARP inhibitor treatments as indicated in the text.

### Growth analysis

The leaves were dissected from the rosette according to their position and ordered in series, photographs were taken at the indicated time points and the leaf area was measured using the ImageJ software for each of the individual leaves. For kinematic analysis the leaves were cleared in 70% Ethanol and mounted with lactic acid on a microscope slide. Afterwards leaves were drawn with a DMLB microscope (Leica) fitted with a drawing tubus. The subsequent cell size and number analysis was done accordingly to [4] and using the leaf pictures and cell drawings to calculate cell size and number per leaf.

### Microarray expression profiling

Labeling, hybridization and raw data collection using the AGRONOMICS1 Affymetrix® Genechips™ were performed at the VIB-Microarray Facility (MAF). Data was analyzed with the bioconductor software [42] for the R statistical environment (www.r-project.org). Quality control and RMA expression estimates were obtained using the affy package [43] whilst the coefficients of differential expression due to 3MB treatment were obtained using the limma package [44]. Expression data is available at ArrayExpress under the accession number (E-MTAB-1466).

### Metabolite profiling

For measuring the polar metabolites GC-TOF-MS as well as LC-TOF-MS were used according to the metabolite platform at the Max-Planck-Institute of Molecular Plant Physiology in Golm [45]. A detailed description of the methods for the LC-MS metabolite profiling has been reported [46]. The chromatogram acquisition was made as described [47]. After import to R the metabolite data was analyzed using the TargetSearch package [48]. The data was further processed by normalization of each metabolite against all values of the same metabolite measured in the same batch and afterwards normalized against the sample median as described in [45].

### Staining

Starch staining was made according to [49], plants were harvested in the end of the subjected 16 h photoperiod, decolorized in hot 80% (v/v) ethanol and stained with a 2% iodine solution. NBT staining was done according to [4], plants were harvest at the end of the subjected 16 h photoperiod, submerged in 0.1% NBT solution for 1 h in complete darkness and afterwards decolorized in hot 80% (v/v) ethanol.

### Flow cytometry

For the flow cytometry analysis (according to 41) nuclei of leaf 2 from 10–12 seedlings were extracted by chopping them with a razor blade in 200 µl of nuclei extraction buffer (Partec). Afterwards they were mixed with 800 µl of DAPI (4,6-diamidino-2-phenylindole) buffer (Partec) with a final DAPI concentration of 1 µg/ml and filtered before the nuclei were analyzed on a CyFlow flow cytometer with the FloMax software (Partec).

### Chlorophyll a fluorescence measurement

The ETR (Electron transport rate) [ETR =  0.5× quantum yield × PAR ×0.84 µequivalents m^−2^ s^−1^], the operating efficiency (quantum yield) of PSII [Y_II  =  (Fm′−F)/Fm′] and the non-photochemical quenching [NPQ  =  (Fm−Fm′)/Fm′] were estimated from an light induction curve using chlorophyll a fluorescence in a MAXI IMAGING-PAM chlorophyll fluorometer (Heinz Walz, GmbH).

### Statistics

Statistical analyses were performed using linear-mixed models using the gls() and lme() functions implemented in the nlme R package (http://www.r-project.org). Where applicable, experiment, block and plate effects were included as random effects and contrasts of interest were made based on treatment and time point.

### Analysis

The GO-term enrichment analysis was made with AmiGO at http://www.geneontology.org/GO [50]. Analysis of diurnal and circadian regulated genes was made based on “***diurnal*** *.* ***mocklerlab*** *.* ***org*** */*” [51]. Visualization of gene expression data was performed using MAPMAN [52].

## Results

Previously it was shown that genetic or chemical down-regulation of PARP leads to an enhanced stress tolerance and growth of plants and plant tissues under unfavorable conditions [5]; [29]. The enhanced growth was mainly associated with higher energy levels, altered ABA signaling and reduced accumulation of defense molecules [5]; [27]; [29]. Nevertheless, the developmental or molecular physiology of how PARP inhibition finally leads to increased growth was not investigated.

**Figure 1:**
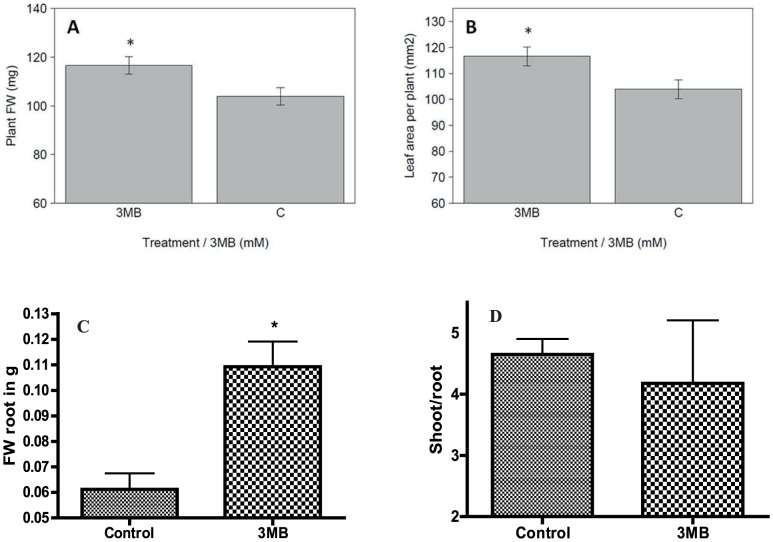
PARP inhibition enhances growth and biomass in control conditions. Hydroponically grown plants were analyzed in respect to leaf area and biomass. 18 plants per experiment and treatment were analyzed, repeated in four experiments and the average leaf area of plants at day 26 (A) or shoot biomass (B), root biomass (C) and the shoot to root ration (D) of plants harvested day 30 is shown. Significant differences (P<0.05) between treated (0.2 mM 3MB) and untreated (125 µl DMSO) plants is indicated by an asterisk.

Previous experiments showed that PARP inhibition could have a positive effect on growth in control conditions when plants were grown on standard agar plates [29]. Since plates also represent a potentially stressful environment, we first sought to establish if growth enhancement occurred under non-stress conditions. We therefore used Arabidopsis plants grown in hydroponics to measure the effects of PARP inhibition with respect to shoot/root biomass, leaf area and shoot to root ratio in control conditions (Figure 1A–D). The addition of the PARP inhibitor 3-Methoxybenzamide (3MB) led to a more than 10% increase in total plant biomass after 30 days of growth, whereas DMSO alone had no significant effects at the used concentration (Figure S1A, S1B). Since we were able to confirm the growth enhancement, we next sought to resolve this response in a temporal manner. We used the agar plate based transfer assay system for this analysis, since all the required tools for kinematic and molecular analysis of plant growth were previously established [4]. Specifically, Arabidopsis Col-0 seedlings were grown for 7 days in control conditions and were afterwards transferred to media containing the PARP inhibitor 3MB or DMSO as control for one, two, four or seven days (day1, day2, day4, day7).

### Chemical PARP inhibition enhances growth of *Arabidopsis thaliana*

**Figure 2:**
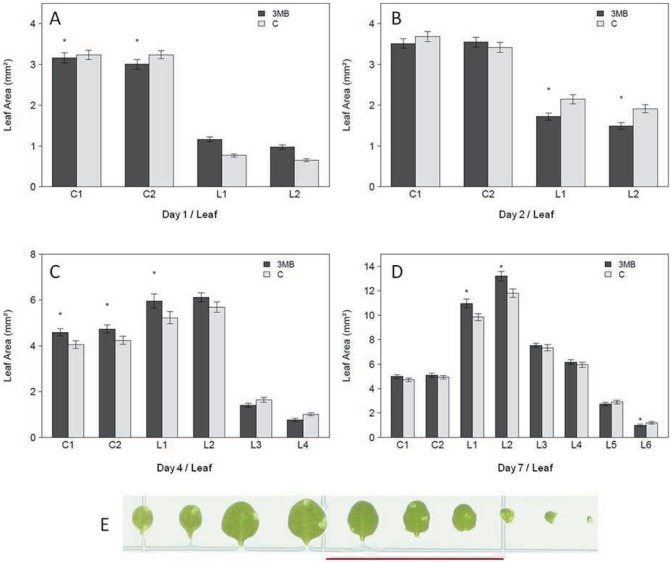
Altered leaf growth in response to PARP inhibition. (A–D) Rosettes harvested at the indicated time points were dissected and the single leaf size was measured by ImageJ with (C) indicating cotelydone and (L) indicating leave. 15–25 seedlings were analyzed in an experiment, repeated in four independent experiments (n = 90). Significant differences (P<0.05) between treated and untreated plants is indicated by asterisks. (E) A typical leaf series of 14 day old plants (day7) is shown. The red bar indicates 2 cm.

The transfer of the plants to PARP inhibitor containing media led to significant effects on growth (Figure 2). Consistent with long-term experiments, a significant enhancement of plant growth was observed by the end of the experiment at 7 days following transfer (Figure 2D). However, at earlier time points (day1 and day2) this assay design allowed us to resolve that PARP inhibition actually led to smaller plants (Figure 2A, B) due to significantly reduced size of both the cotyledons (C) and the first two true leaves (L). However, this was quickly reversed so that at later time points the opposite effect was observed, with increased growth of the plants driven mainly by leaf one and two (Figure 2C–D). Interestingly, the analysis of individual leaves also showed a consistent pattern of leaf size being first reduced then later enhanced also for the subsequently appearing leaves 3 and 4. Overall these data indicate that PARP influences growth and development particularly of young leaves.

### Kinematic analysis of growth

**Figure 3:**
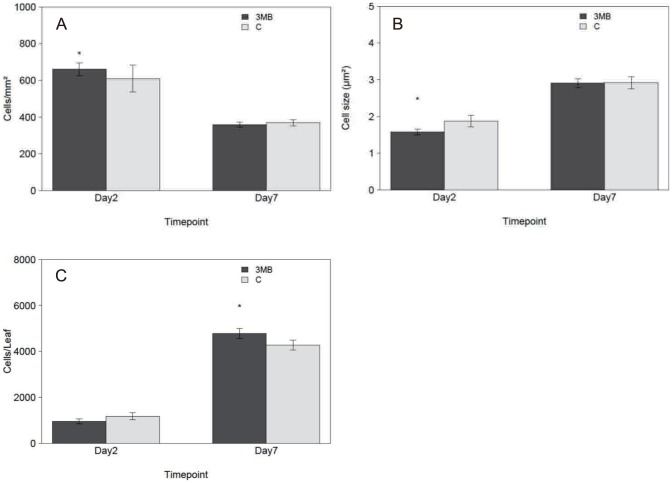
PARP inhibition induced cell number increase. Arabidopsis leaf two was analyzed after two days of transferring the seedlings (day2) or after seven days (day7) either to treatment or control conditions. (A) Cell number per mm^2^, (B) cell size in µm and (C) the calculated cell number per leaf is presented. Seedlings were previous grown for seven days in control conditions. In each experiment 4–6 leafs were analyzed and the experiment repeated three times independently (n = 12–18). Significant differences (P<0.05) between treated and untreated plants is indicated by an asterisk.

**Figure 4:**
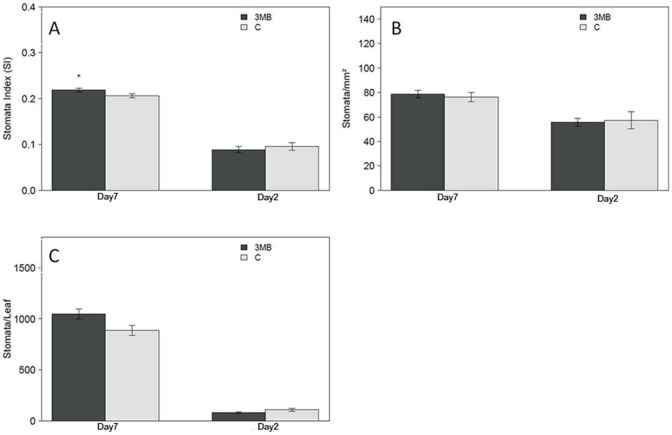
PARP inhibition and its effect on guard cell development. Arabidopsis leaf two was analyzed after two days of transferring the seedlings (day2) or after seven days (day7) either to treatment or control conditions. (A) The stomata index (SI) is shown, calculated by number of stomata divided by number of cells, (B) the number of stomata per mm^2^ and (C) the calculated total number of stomata per leaf is presented. In each experiment 4–6 leafs were analyzed and the experiment repeated three times independently (n = 12–18). Significant differences (P<0.05) between treated and untreated plants is indicated by an asterisk.

Since it underwent the strongest changes in response to PARP inhibition during the timing of this experiment, leaf two was selected for detailed cellular analysis. As leaf size phenotypes were strongest at day2 and day7 these time points were chosen for more detailed investigations. Results clearly showed that PARP inhibition increased cell number (Figure 3A). At day2 the number of cells per mm^2^ was higher (∼10%) for the PARP inhibited plants and at the same time those cells were smaller (Figure 3B), being approximately 80% of the control. This lowered cell size causes the observed reduced leaf area at day2. This changed when day7 was analyzed, the cells of both treated and untreated seedlings were now equal in size leading to a similar number of cells per mm^2^ but as size is equal the increased leaf growth is caused by the increase in cell number (Figure 3C). The increased cell number is the basis for the observed enhanced growth of PARP inhibited plants. Furthermore, the cellular analysis showed that stomatal development was also responsive to PARP inhibition. The number of stomata increased relative to the total cell number more than expected based on the increased leaf area at day7. In other words, the second leaf had a significantly higher stomata index, indicating that the number of stomata relative to the total cell number is increased (Figure 4). In summary, the leaf size and cell analysis revealed a clear temporal pattern of PARP inhibition affecting cell growth and leaf size. The experiments showed that the finally observed leaf size increase was due to increased cell number. For further investigation cell cycle progression was monitored by analyzing ploidy levels.

### Cell cycle analysis

**Figure 5:**
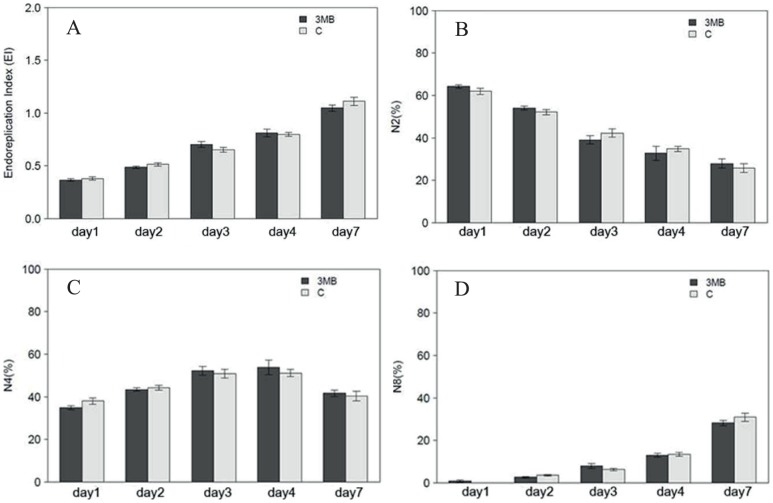
Ploidy analysis of leaf two. Arabidopsis leaf two was analyzed at the indicated time points from plants subjected to PARP inhibition or control conditions. (A) the calculated EI is shown, EI represents the number of endo cycles undergone by a typical nuclei (EI  =  1*4N+2*8N+3*16N), in (B–D) percentage of 2N, 4N and 8N nuclei is presented. 10–12 leaf two were pooled for a replicate, four biological replicates were analyzed in each of the two independent experiments (n = 8).

Increased cell number in Arabidopsis leaves can arise through either prolonged phase of cell division linked to late entry into cell expansion or through faster division cycles. To distinguish between the two possibilities cell ploidy levels were tested. The switch from cell division into expansion coincides with onset of endoreduplication and so if the first scenario was correct then later onset of endoreduplication in the treated plants would be observed [53]; [54]. Therefore, as a measurement of the genome copy number [41], the ploidy level for both treatments was measured at each time point and the endoreduplication index calculated (Figure 5A). An additional time point at day 3 was included for optimal coverage of the transition phase between reduced and enhanced growth. However, no differences in the endoreduplication index or in the ploidy level (Figure 5B–D) could be observed at any time point during the experiment between seedlings grown in control conditions and those exposed to the PARP inhibitor. In summary, the experiments showed that PARP inhibition induced growth is driven by enhanced cell number related to shortening of cell division cycle resulting in more divisions and more cells.

### PARP as a regulator of gene expression

**Figure 6:**
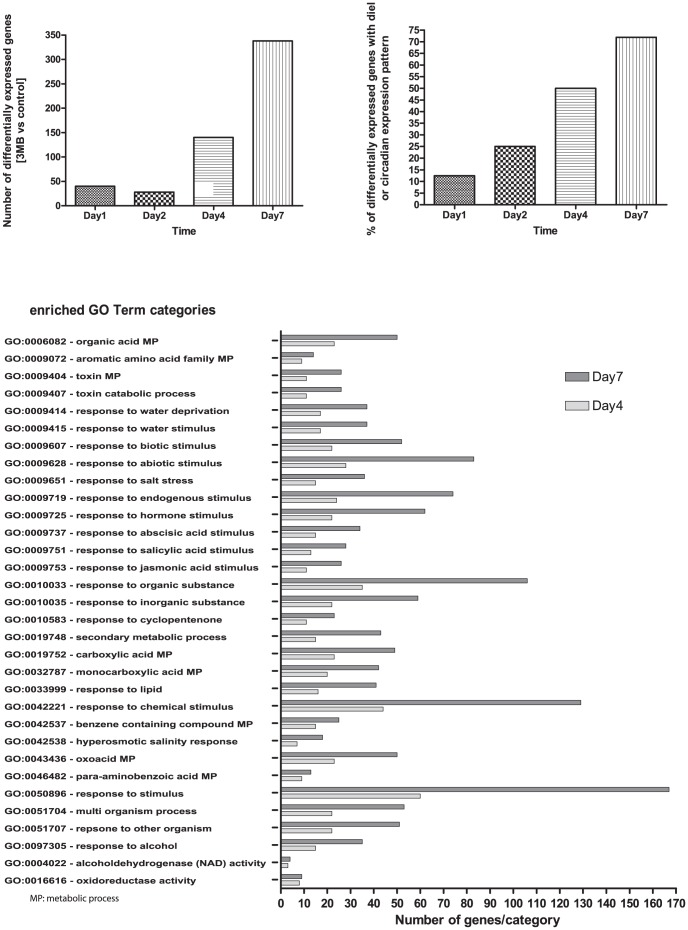
Gene expression analysis. The transcription profile of leaf two after transfer either to treatment or control conditions was investigated by microarrays at the indicated time points. 10 leaf two from each of the five biological replicates within a single experiment were pooled and subjected to analysis, this was repeated in three independent experiments (n = 3). (A) shows the total number of differentially expressed genes, based on a log2 difference, (B) shows the percentage of the differentially expressed genes which show circadian or diurnal expression pattern and (C) presents the clustering of the differentially expressed genes by GO-term enrichment analysis, significantly enriched GO-term categories are presented.

**Figure 7:**
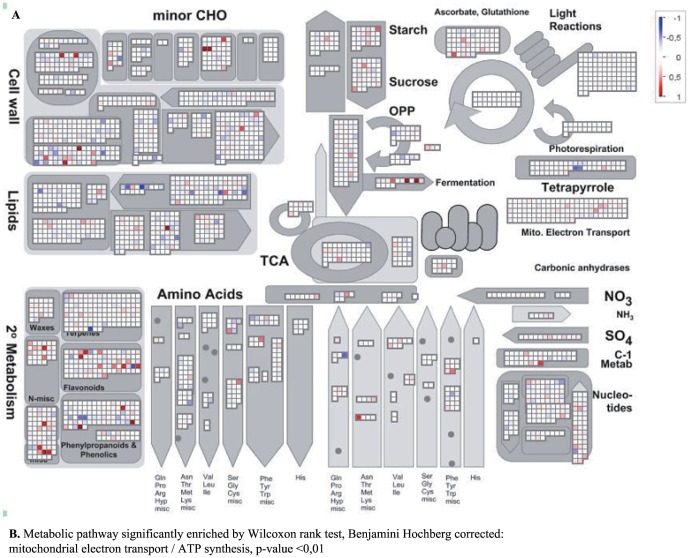
MAPMAN metabolic overview of transcriptional changes day4. Shown are the overall gene expression changes related to the metabolism day4 between 3MB treated leaves 2 and controls. The Wilcoxon rank sum, with Benjamini-Hochberg correction, is shown below.

**Figure 8:**
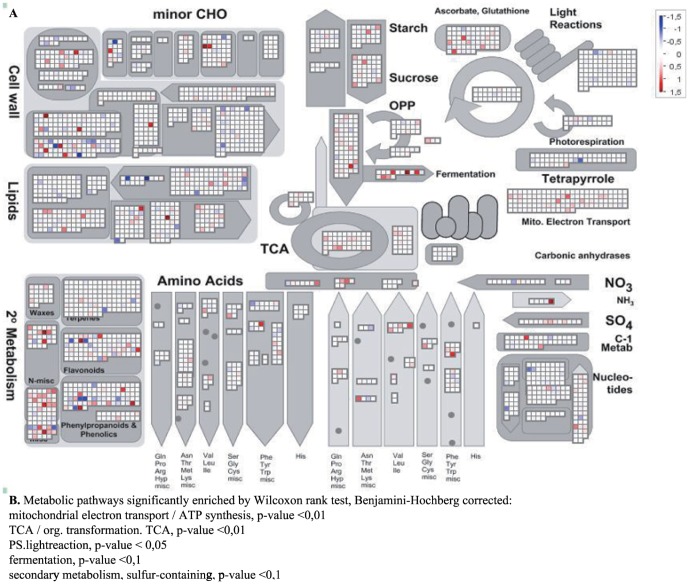
MAPMAN metabolic overview of transcriptional changes at day7. Shown are the overall gene expression changes related to the metabolism day7 between 3MB treated leaves 2 and controls. The Wilcoxon rank sum, with Benjamini-Hochberg correction, is shown below.

Previous transcriptional analysis of PARP inhibited plants had focused on the whole plant level and/or long-term genetic or chemical inhibition of PARP [5]; [27]; [29] and thus provide little insight into changes associated with growth enhancement under non-stress conditions. Therefore, to gain more insights into PARP function and its effect on development and growth, the transcriptome of the second leaf parallel to the one used for kinematic analysis was analyzed. Using the same leaf as for the detailed cellular analysis enabled a tight and temporally resolved link between the transcriptome and the observed physiological changes. These data show that PARP inhibition changes the expression of a few hundred genes, but does not influence the expression of PARP or Poly(ADP-ribose)glycohydrolase (PARG) even 7 days after transfer (Figure S2). The analysis revealed 40, 28, 140 and 338 genes that were changed at least 2-fold after one, two, four and seven days, respectively (Figure 6A). Cross time point analysis shows that an increasing percentage of the deregulated genes have a diel or circadian expression pattern ∼10% at day1 up to ∼70% at day7 (Figure 6B). The larger number of deregulated genes at day4 and day7 enabled a GO-term enrichment analysis. This analysis revealed a significant over-representation of genes responsive to stimuli, abiotic stress, hormones such as jasmonic acid (JA) or ABA, secondary metabolites and lipids (Figure 6C). Nevertheless, the group of deregulated genes does not include typical cell cycle or developmental genes such as *CDKA*'s or *KRP*'s [55]; [56]. However, important regulators of growth and metabolism (Figure 7), more specifically *MYB4* (At4G38620) [57] or a ADP-ribosylation factor (At2G15310) [58]; [59] were induced at multiple time points. At the last time point the highest number of transcriptional changes was observed and analysis revealed a strong over-representation of secondary metabolism, particularly phenylpropanoid pathway genes as well as genes involved in energy metabolism (Figure 8). At the same time genes related to photosynthesis such as *ELIP1* (At3G22840), *ELIP2* (At4G14690) are induced (Table S1) while low energy status markers such as *DIN2* (At3G60140) and *DIN6* (At3G47340) were unaltered suggesting a continuous and normal energy status despite the enhanced growth and efficient photosynthesis in PARP inhibited plants. This is supported by the observation that the effective photosynthetic quantum yield (Y_II), as well as the electron transport rate (ETR) are significantly increased upon PARP inhibition (Figure S3).

In summary the expression profiling revealed that the number of deregulated genes increased over time and that a small subset of genes responded at multiple time points to PARP inhibition. Deregulated genes often had circadian or diel expression pattern and encode proteins interacting with the cell cycle and proteasome as well as known growth regulators and markers of the metabolic status.

### PARP inhibition changes primary metabolism

Growth and metabolism are closely connected and PARP inhibition significantly enhances growth. Therefore, to gain more insights into the molecular effects of PARP inhibition metabolite profiling was performed. On the basis of technical limitations we used the shoot comprising all rosette leaves, which was further justified by the physiological analysis showing PARP inhibition enhanced overall shoot growth. The results showed that PARP inhibition led to changes in the level of metabolites in various compound classes such as amino acids, organic acids or fatty acids (Table 1). In total 63 different known compounds were identified in all samples (n = 120), of which 40 showed a significant change for at least one of the four time points (Table S2). Among these changes we were interested to identify metabolites that showed a consistent or coordinated patterns of up- or down-regulation in response to PARP inhibition. We found that Leucine was down-regulated day1 but was consistently up-regulated at all other days, while histidine, tyrosine, asparagine and beta-alanine all showed the opposite effect.

**Table 1:** PARP activity and effects on primary metabolism.

Amino acids and derivates	Day1	Day2	Day4	Day7
Aspartate	***0.78***	*0.95*	***0.35***	*1.10*
Asparagine	***3.33***	***1.38***	*0.99*	*0.42*
Threonine	***0.83***	*1.03*	*1.26*	*1.07*
Pyroglutamat	***0.72***	*2.16*	*1.09*	***1.43***
Ornithine	*1.35*	***1.96***	*0.45*	*1.11*
Proline	*0.91*	*0.82*	*0.96*	***0.32***
Spermidine	*0.81*	***0.58***	*0.95*	*0.97*
Tyramine	*0.84*	*0.85*	*0.91*	***1.33***
Tyrosine	***2.55***	*1.48*	*1.07*	*0.64*
Serine	*0.78*	***1.42***	*1.39*	*1.06*
Glycine	*1.03*	*0.96*	***1.73***	*0.86*
beta_alanine	***3.38***	*0.89*	***0.50***	***0.22***
Alanine	*0.96*	***1.23***	*1.04*	*0.95*
Histidine	*1.20*	*0.88*	*0.66*	***0.43***
Leucine	***0.83***	*1.37*	*1.47*	***1.54***
**Organic acids**				
Pyruvic acid	***0.69***	*1.00*	*0.67*	*1.12*
Fumaric acid	***0.74***	*0.91*	*0.87*	***1.56***
Malic acid	***1.59***	*0.75*	*1.56*	*0.94*
2-Oxoglutarat	***0.60***	*1.14*	*1.20*	*1.10*
4-Aminobutyrat	*0.90*	*0.91*	***0.57***	***0.60***
Ribonic acid	*0.86*	***0.35***	***0.33***	***0.42***
Shikimic acid	***0.67***	*0.84*	*0.73*	***1.35***
**Fatty acids**				
Glycerol	***0.23***	***0.58***	***0.57***	*1.33*
Docosanoic acid	***0.50***	*0.82*	*0.61*	*0.41*
Hexacosanoic acid	***0.55***	*0.97*	*1.32*	*1.01*
Octadecanoic acid	***0.62***	*0.87*	*1.18*	*1.41*

The metabolite profile of Arabidopsis shoots was analyzed by GC-MS at the indicated times following transfer of the seedlings after 7 days growth on control media to either control or PARP inhibitor containing media. Plants were grown in parallel to those for the physiological and microarray analysis. Five individual samples, each a pool of 10–12 plants, were harvested and analyzed in three independent experiments (n = 15). Amino, organic and fatty acids with significant changes in their relative abundance are shown. Metabolite content relative to the (−3MB), with red and blue indicating accumulation and depletion, are shown.

Beside amino acids and precursors, carbon metabolism is a central aspect of primary metabolism and we therefore wanted to understand in more detail the consequences of PARP inhibition. Over the time course a clear up-regulation of fumarate and shikimate could be seen at day7. With respect to fatty acids it is noteworthy that changes were only seen directly after transfer at day1, when a massive down-regulation was observed along with an accumulation of pre-cursors in 3MB treated plants.

Finally, we were interested to investigate the impact of PARP inhibition on the amount of NAD+ breakdown products, whereby nicotinate - a product of the NAD+ degradation and salvage pathways derived from Nicotinamide which is a direct product of PARP mediated NAD+ cleavage – attract our attention. The GC-MS measurement revealed hereby a significant reduction of nicotinate day2 and at all later time points to 1/3 of the control plants (Table S2), consistent with a reduction in the production of nicotinamide by PARP.

### PARP activity regulates secondary metabolism

**Figure 9:**
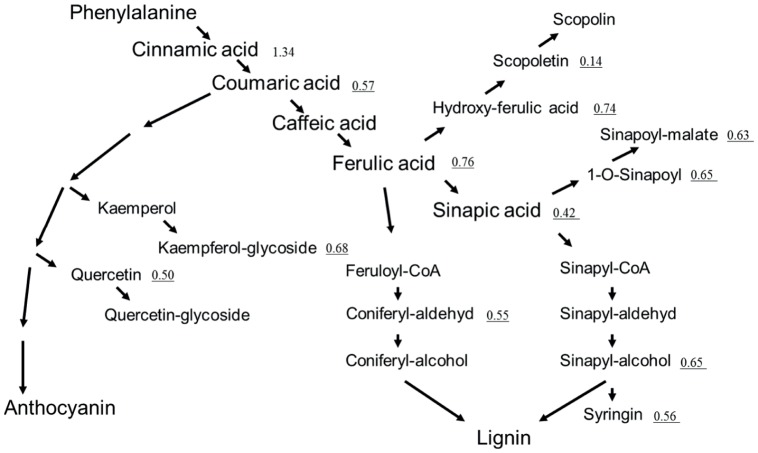
PARP inhibition and its effect on the phenylpropanoid pathway. Arabidopsis shoots were harvested at day7 after transfer. Per replicate 10–12 seedlings were harvested, with 5 biological replicates in each of the three independent experiments (n = 15). The metabolite content relative to the control (no 3MB) is shown and when changed significantly the relative content is underlined. Only metabolites with a clear annotation and positioning within the pathway are shown.

PARP activity has a significant effect on the regulation of secondary metabolism, particularly anthocyanin accumulation under stress [29]. To explore if this holds true in control conditions, the consequences of PARP inhibition on secondary metabolism potentially influencing growth was investigated by LC-MS analysis according to [46] with samples harvested on day7. Besides 10 diverse compounds showing either up- or down-regulation (Table 2) the analysis revealed that PARP inhibition in particular influences the phenylpropanoid pathway. PARP inhibited plants showed an overall reduction of metabolites related to this pathway (Figure 9), in total 23 of those metabolites were depleted in their relative abundance (Table S3). Significant reduction of metabolite levels was observed in all branches of the phenylpropanoid pathway including the flavonols and the lignin branch, with an average reduction of ∼50%. This reduction was not observed for the precursor shikimic acid and the early appearing metabolite cinnamic acid. Another interesting question was if PARP inhibition had an impact under non-stress conditions on redox metabolites? The LC-MS analysis showed that glutathione content is reduced while in contrast its precursor gamma-glutamyl significantly increased compare to control plants. Ascorbic acid was about half the level of the control in PARP inhibited plants, whilst at the same time NAD+ content was found to be unchanged (Table 3). The unchanged amount of energy rich compounds holds also true for starch for which no difference could be observed via iodine staining (Figure S4). Summarizing the metabolite analysis, PARP inhibition influences primary and secondary metabolism under non-stress conditions. It was demonstrated that among primary metabolites amino and fatty acids levels are most sensitive to changes in PARP activity, whilst for secondary metabolism effects were particularly strong with respect to the phenylpropanoid pathway.

**Table 2:** PARP inhibition altering secondary metabolism.

3MB/control	Compound class	KNAPSACK Synonym	5-top
91.15	vitamin B6 metabolism	C00007509: Pyridoxal	up
13.49	Amino acid metabolism	C00001401: p-Aminobenzoic acid| C00007382: Anthranilic acid	up
2.98	IAA derivate	C00000124: 4-O-(Indole-3-acetyl)-D-glucopyranose	up
1.83	Glutathione metabolism	C00007507: L-gamma-Glutamyl-L-cysteine	up
1.62	Amino acid metabolism	C00000113: Indole-3-carboxylic acid	up
0.42	Phenylpropanoid biosynthesis	C00034327: (E)-Sinapic acid	down
0.40	Putative Flovonoid	C00004565: Nimbecetin	down
0.38	Carotenoid biosynthesis	C00003787: Violaxanthin	down
0.35	IAA derivate	C00007573: 6-hydroxyindole-3-carboxylic acd 6-O-beta-D-glucopyranoside	down
0.16	Phenylpropanoid biosynthesis	C00002499: Scopoletin	down

Arabidopsis shoots were harvested at day7 after transfer. Per replicate 10–12 seedlings were harvested, with 5 biological replicates in each of the three independent experiments (n = 15) and analyzed using LC-MS. The five strongest up- and down-regulated metabolites are shown.

**Table 3:** PARP inhibition affects redox metabolite concentrations.

Compound	3MB/control	P-value
Glutathione	0.8	0.001
Gamma-glutamycysteine	1.83	0.008
Ascorbic acid	0.43	0.007
NAD+	0.95	0.681

Arabidopsis shoots were harvested at day7 after transfer. Per replicate 10–12 seedlings were harvested, with 5 biological replicates in each of the three independent experiments (n = 15) and analyzed using LC-MS. The relevant data of the redox metabolites was extracted from the LC-MS data set.

Overall, the experiments revealed a time dependent response of Arabidopsis to PARP inhibition on leaf growth and cell number. The increased growth is based on more cells and this was underpinned by transcription and metabolite changes associated with different forms of growth regulation.

## Discussion

We present a successful approach to enhance growth of plants under non-stress conditions. The approach is based on the chemical inhibition of PARP activity, a method originally described as a tool to enhance abiotic stress tolerance [14]; [5]. Our experiments showed that PARP inhibition changes the growth of plants rapidly by increasing the cell number in the leaves. Increased cell number as a basis for enhanced growth is in line with previous reports showing that a higher number of cells drive growth, rather than altered cell size [4]; [60].

The observed altered cell size at day2 is not caused by genome copy number changes, as indicated by the unchanged endoreduplication index and ploidy levels, or other processes such as compensated cell enlargement [61]; [62]. That compensatory processes are not relevant is further supported by the equal cell size at day7. Increased cell number is at least partially driven by the increase in guard cell number indicated by the increased stomata index. As SI can continue to rise even once all pavement cells are completely expanded [4], this may indicate a prolonged effect of PARP inhibition on meristemoids, the stomatal precursor cells. This increase in guard cell number could also potentially explain why increased cell number is not leading to increased ploidy levels as guard cells show a rather constant low ploidy level [63]; [64]. Growth based on more cells would go together with increasing ploidy level. The increase in cell number is supported by enhanced transcription of cell cycle interacting proteins such as DNAJ or F-Box kelch repeat domain encoding genes. Two out of three changed DNAJ (At3G13310, At2G17880) and four out of five changed F-Box kelch-repeat domain proteins (At2G44130, At1G23390, At3G59940, At3G61060) observed at day4 and day7 are able to interact with anaphase promoting complex and proteasome associated proteins such as APC8/CDC23 and SKP-like2 [65]. Both processes and protein families were recently highlighted for having great impact on cell cycle progression [66]; [67]. Another interesting observation is the induction of *ADP-ribosylation factor (ARF)* expression, observed at the time of enhanced growth, day 4 and day7. Over-expression of a single ARF gene from wheat in Arabidopsis enhances growth significantly under non-stress conditions [59], whereas down-regulation inhibits growth [58]. The observed combinatorial over-expression of *DNAJ* (At3G13310), *F-Box* (At2G44130) and *ARF* (At2G15310) genes might therefore be sufficient to drive the enhanced leaf growth.

It is also worth to consider the involvement or interaction with other pathways. For example, changes in the redox balance were observed and could be another important regulatory factor, since glutathione availability strongly influences growth and as PARP activity and nuclear glutathione accumulation are associated with each other [68]; [39]. On the other hand, the enrichment of ABA, JA and SA responsive genes indicates that PARP inhibition and the resulting growth enhancement could also be accompanied by changes in the hormone status. Alternatively, the reduced metabolite content of the phenylpropanoid pathway might lead to the observed changes particular for JA as indicated by enhanced transcription of JA-responsive genes in *tt5* mutants [69]. The overall hormone changes are in agreement with previous results showing a strong interaction of PARP inhibition and changes in ABA responsive gene expression [27] and are in addition to the postulated interplay of PARP and SA related defense mechanism [31]. However, we did not find specific evidence this contributes to the growth enhancement. In this respect it is worth mentioning that structural analogs of 3MB such as Imidacloprid (IMI), a neonicotinoid, have been consistently associated with enhanced crop performance under stress conditions potentially by interacting with the SA-pathway [70]. Structural analogies raise the possibility that either IMI or its metabolized derivatives may interact with PARP or whether there is an interaction with the NAD+/SA-pathway leading to the reported growth enhancement and stress protection [71].

Enhanced growth certainly requires sufficient support of energy, lipids and cell wall material. The fact that energy supply was sufficient is indicated by the normal transcription of *DIN2* and *DIN6*, both considered to be marker genes of low energy status when induced [72]; [73]; [6]. This is in line with previous results showing that PARP inhibition enhances the energy content of plants, in particular ATP and NAD+ levels [5]; [29]. Besides PARP inhibition and reduced NAD+ consumption, enhanced growth needs further energy supply which ultimately depends on photosynthesis. The transcriptional analysis of leaf two demonstrates that photosynthesis related genes such as *ELIP*'s are up-regulated. The protective role of these genes for photosystems [74] may explain the enhanced effective quantum yield of PARP inhibited plants [29] and the observed increase in electron transport. Alternatively, or in addition, the increased number of stomata could contribute to energy supply via enhanced CO_2_ uptake and carbon fixation.

It appears that the enhanced growth in general is not strongly reflected in terms of any overall increase in primary metabolites or of organic acids in particular, likely since they are immediately utilized for growth. However, in the regulatory context several relevant changes were observed. Firstly, the increase in leucine content is notable since it has been implicated as a potential metabolic activator of signaling processes [75] and the increasing content is temporally related to the increasing number of altered transcripts and enhanced growth. In addition a constant increase in the sucrose content was observed throughout the experiment, concomitant with the unaltered expression of energy status marker genes including *DIN2* and *DIN6* despite enhanced growth. Sucrose is discussed to be a major integrator and signal involved in growth regulation [76]; [77], particularly in controlling cellulose and starch biosynthesis which are both important components of biomass production [8]; [78]. Photosynthesis, sucrose, starch production and finally growth are all driven by the availability of light and are under the control of circadian and diel regulation. For example, carbohydrate availability is controlled by the circadian clock and correct anticipation of the day is required for optimal growth by controlling photosynthesis and thereby energy production [79]; [80]. The potential interaction between PARP and the circadian clock is indicated by the increasing number of deregulated genes which are circadian regulated. Since it was previously shown that enhanced PAR level can lead to strong shifts in circadian rhythms [32] it is tempting to speculate that PARP interacts with the circadian clock. Together with the observed changes in the primary metabolism it is conceivable that PARP inhibition is modulating carbohydrate and energy production in a way favorable for overall growth and biomass production.

An overall reduction of phenylpropanoid related metabolites at the time of enhanced growth was observed, while at the same time the precursor shikimic acid is increased but also unchanged amounts of cinnamic acid the second compound of the phenylpropanoid pathway were detected. This means that the initial reaction of Phenylalanine-ammonium lyase (PAL) is unaffected, despite observing a 50% reduction in the expression of *PAL1* at day7. This is supported by previous findings showing that low concentrations of chemical PARP inhibitors as used in our study are not affecting PAL activity [81]. In that respect it is noteworthy, that the transcription profiling revealed enhanced expression of the *Myb4* (At5G38620) gene shown to be an important negative regulator of different steps downstream of PAL in the phenylpropanoid pathway [57]; [82]. Based on the reduced metabolite levels and the enhanced expression of the negative regulatory transcription factor MYB4, it is tempting to speculate that PARP might regulate MYB4 leading to the observed changes in secondary metabolism. Alternatively, the reduced glutathione and ascorbic acid content potentially regulate the accumulation of phenylpropanoid pathway related metabolites negatively. This would be in line with previous results showing that low glutathione as well as low ascorbate leading to low anthocyanin content under stress [83]–[85]. This link to other redox metabolites than NAD+ was also observed in experiments with reduced PARP activity [29].

The diverse consequences of PARP inhibition described above raise the question of the specificity of 3MB as PARP inhibitor. Previous studies [86] showed that benzamides had only little influence on mono(ADP-ribose)transferases and that 3MB is a highly potent PARP inhibitor. Nevertheless, chemical PARP inhibitors may trap PARP at single-strand breaks (SSB) and as consequence also inhibit other SSB related proteins, which need PAR for their activity [87]. The used low dose of 3MB was compared against genetically modified plants with no detected difference in performance and the effect of chemical and genetic inhibition was not additive [29]. Furthermore, in biochemical tests no adverse effect of such low inhibitor dose was found [81]. We thus believe that the effects presented here are likely to be based on the reduced activity of PARP itself.

As discussed above PARP activity is involved in the regulation of the cell cycle and/or development by influencing a particular subset of genes, redox as well as energy homeostasis, primary and secondary metabolism finally leading to enhanced growth. The presented transcriptional changes are connected with the observed physiological and metabolic changes. The presented data provides new insights into plant growth regulation, shows that PARP is a prominent player within growth regulating circuits and opens interesting new starting points for biotechnological modulation of growth.

## Supporting Information

Figure S1 **Used DMSO concentrations showing no impact on Arabidopsis.** Representative pictures of Arabidopsis seedlings grown hydroponically for 14 days (A) or 26 days (B) with or without DMSO are shown.
(EPS)

Figure S2 **PARP inhibition is not influencing** ***PARP*** **or** ***PARG*** **expression.** *PARP* and *PARG* expression shown is comprised from the within this work generated expression analysis data [E-MTAB-1466]. The number of days (D) by which the seedlings were subjected to PARP inhibiting (+) or control (−) conditions is indicated.
(EPS)

Figure S3 **PARP inhibition and photosynthesis.** The, photosynthetic yield (Y_II) (A), non-photochemcial quenching (NPQ) (B) and ETR (Electron transport rate) (C) is presented of Arabidopsis seedlings grown either for eight days in control (−3MB) or in treatment (+3MB) conditions. 8–18 seedlings were analyzed in each of the four independent experiments (n = 42). A significant difference (P<0.05) between the conditions is indicated by an asterisk.
(EPS)

Figure S4 **PARP inhibition is not changing starch accumulation.** Representative pictures of Arabidopsis seedlings stained with iodine solution day1, day2 or day7 after transfer to either 3MB containing (A) or control plates (B).
(TIF)

Table S1 **Overview of differentially expressed genes.** The transcription profiles of *Arabidopsis* leaf two were analyzed via AGRONOMICS1 microarrays at the indicated time points of leaves 2 from seedlings transferred after 7 days of growth on control media either to control or PARP inhibitor containing media. Shown are all genes with changes in gene expression with the direction of change at the different time points relative to the (−3MB) control. Samples comprised of pools of 50 leaves taken from 10 plants of each of the five replicates within a single experiment, this was repeated in three independent experiments (n = 3). Direction of change is indicated by red - induction and blue – repression, genes which changed the time point before are marked in bold.
(PDF)

Table S2 **Complete list of detected primary metabolites.** For the GC-MS analysis samples were taken in parallel to samples for the physiological measurements for both conditions (+/− 3MB) at day1, day2, day4 and day7. The experiment was repeated three times independently with five replicates each (n = 15). Relative abundance of metabolite in the 3MB treated sample compared to the control samples are shown.
(PDF)

Table S3 **Complete list of detected secondary metabolites.** For LC-MS metabolite profiling 5 replicate samples were taken in parallel to samples to the GC-MS for both conditions (+/− 3MB) seven days after transfer (day 14) in three independent experiments (n = 15). Metabolites with significant changes in their relative abundance in the PARP inhibited plants are shown in bold. Individual peak number, the chemical sum formula of the detected compound and the KEGG as well as Knapsack annotation were shown.
(XLSX)
