# Supplementary figures and images for: Poly(ADP-Ribose)Polymerase Activity Controls Plant Growth by Promoting Leaf Cell Number
Source: PLoS One. 2014 Feb 28;9(2):e90322. doi: 10.1371/journal.pone.0090322 (PMC3938684; doi:10.1371/journal.pone.0090322)

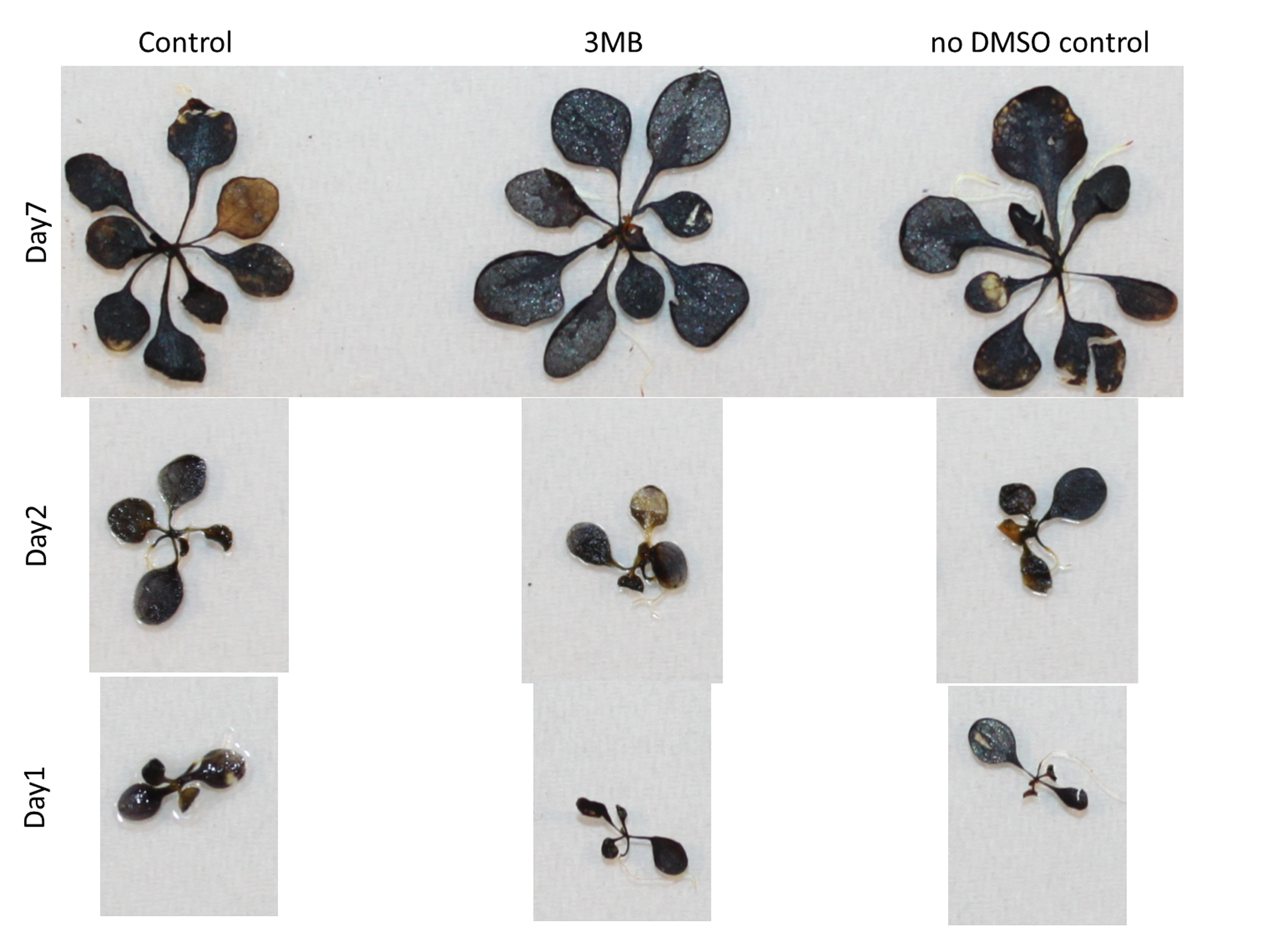

Supplement: Figure S4 — PARP inhibition is not changing starch accumulation. Representative pictures of Arabidopsis seedlings stained with iodine solution day1, day2 or day7 after transfer to either 3MB containing (A) or control plates (B). (TIF) [file pone.0090322.s004.tif]
