# Supplementary material for: Poly(ADP-Ribose)Polymerase Activity Controls Plant Growth by Promoting Leaf Cell Number
Source: PLoS One. 2014 Feb 28;9(2):e90322. doi: 10.1371/journal.pone.0090322 (PMC3938684; doi:10.1371/journal.pone.0090322)
Supplement: Table S1 — Overview of differentially expressed genes. The transcription profiles of Arabidopsis leaf two were analyzed via AGRONOMICS1 microarrays at the indicated time points of leaves 2 from seedlings transferred after 7 days of growth on control media either to control or PARP inhibitor containing media. Shown are all genes with changes in gene expression with the direction of change at the different time points relative to the (−3MB) control. Samples comprised of pools of 50 leaves taken from 10 plants of each of the five replicates within a single experiment, this was repeated in three independent experiments (n = 3). Direction of change is indicated by red - induction and blue – repression, genes which changed the time point before are marked in bold. (PDF) [file pone.0090322.s005.pdf]

Transcriptional log2 fold change

| | |
|---|---|
| AT3G15600 | 1.59 |
| AT3G13310 | 1.59 |
| AT1G26796 | 1.55 |
| AT2G03565 | 1.36 |
| AT4G01245 | 1.34 |
| AT4G14149 | 1.33 |
| AT4G07917 | 1.33 |
| AT1G43050 | 1.32 |
| AT1G70860 | 1.30 |
| AT5G18748 | 1.30 |
| AT4G04273 | 1.28 |
| AT3G61962 | 1.26 |
| AT3G61723 | 1.26 |
| AT2G30766 | 1.25 |
| AT4G08078 | 1.24 |
| AT2G10120 | 1.22 |
| AT3G54925 | 1.21 |
| AT5G47818 | 1.17 |
| AT1G04684 | 1.16 |
| AT5G64401 | 1.14 |
| AT3G22231 | 1.14 |
| AT5G41401 | 1.14 |
| AT1G29410 | 1.13 |
| AT2G44130 | 1.08 |
| AT3G46086 | 1.08 |
| AT4G31136 | 1.06 |
| AT1G33300 | 1.04 |
| AT1G05730 | 1.01 |
| AT5G45573 | 1.01 |
| AT3G43526 | -1.00 |
| AT1G27540 | -1.01 |

| | |
|---|---|
| **AT3G13310** | **1.83** |
| AT2G44130 | 1.75 |
| AT3G56980 | 1.64 |
| **AT1G26796** | 1.42 |
| AT2G11840 | 1.40 |
| AT1G46048 | 1.31 |
| AT1G50780 | 1.30 |
| AT1G47786 | 1.25 |
| AT2G41240 | 1.24 |
| AT2G27228 | 1.20 |
| AT5G34795 | 1.19 |
| AT3G27809 | 1.14 |
| AT5G38005 | 1.10 |
| AT4G26005 | 1.08 |
| AT1G74448 | 1.07 |
| AT1G47400 | 1.03 |
| AT4G30140 | 1.03 |
| AT4G00380 | 1.03 |
| AT5G39550 | 1.02 |
| AT2G04041 | 1.01 |
| AT1G33550 | 1.01 |
| AT1G23810 | -1.11 |
| AT2G23321 | -1.11 |
| AT2G05510 | -1.12 |
| AT1G71370 | -1.13 |
| AT3G50682 | -1.25 |
| AT3G42450 | -1.37 |

| | |
|---|---|
| AT3G02480 | 3.46 |
| **AT3G13310** | **3.46** |
| **AT2G44130** | **3.30** |
| AT5G59310 | 3.22 |
| AT4G12580 | 2.83 |
| AT1G52690 | 2.77 |
| AT5G59320 | 2.72 |
| AT1G05680 | 2.26 |
| AT2G14610 | 2.25 |
| AT3G04000 | 2.15 |
| AT4G10860 | 1.99 |
| AT2G33380 | 1.98 |
| AT2G23170 | 1.95 |
| AT2G29350 | 1.90 |
| AT5G06760 | 1.90 |
| AT3G57260 | 1.88 |
| AT3G22840 | 1.87 |
| AT1G10585 | 1.83 |
| AT1G17170 | 1.78 |
| AT5G62162 | 1.76 |
| AT2G37770 | 1.74 |
| AT2G43570 | 1.66 |
| AT2G47770 | 1.65 |
| AT2G43871 | 1.65 |
| AT2G02990 | 1.64 |
| AT1G05340 | 1.62 |
| AT2G29490 | 1.56 |
| AT1G53625 | 1.56 |
| AT3G44300 | 1.51 |
| AT5G66400 | 1.50 |
| AT3G17520 | 1.50 |

| | |
|---|---|
| **AT3G13310** | 4.41 |
| **AT2G44130** | 4.01 |
| AT1G69880 | 3.73 |
| **AT3G04000** | 3.47 |
| **AT2G02990** | 3.31 |
| **AT4G12580** | 3.18 |
| **AT2G23170** | 2.92 |
| AT3G44350 | 2.86 |
| **AT4G14690** | 2.76 |
| **AT1G77120** | 2.67 |
| **AT4G34135** | 2.38 |
| **AT1G17170** | 2.34 |
| **AT1G63840** | 2.32 |
| AT1G19020 | 2.28 |
| **AT3G22840** | 2.26 |
| **AT1G10585** | 2.18 |
| **AT3G02480** | 2.13 |
| AT2G38380 | 2.12 |
| AT3G53232 | 2.11 |
| AT1G66570 | 2.11 |
| **AT4G10860** | 2.09 |
| **AT5G59310** | 2.07 |
| **AT3G10912** | 2.07 |
| **AT1G05680** | 2.07 |
| **AT1G75900** | 2.07 |
| AT1G66570 | 2.04 |
| **AT4G38620** | 1.98 |
| AT2G23110 | 1.97 |
| AT2G29500 | 1.96 |
| AT1G66570 | 1.95 |
| AT4G12480 | 1.92 |

| | |
|---|---|
| AT4G35025 | -1.02 |
| AT4G37810 | -1.05 |
| AT5G64541 | -1.15 |
| AT4G06496 | -1.16 |
| AT5G40981 | -1.16 |
| AT5G15254 | -1.23 |
| AT1G15910 | -1.30 |
| AT5G27967 | -1.31 |
| AT4G05494 | -1.37 |

| | |
|---|---|
| AT1G01115 | 1.47 |
| AT4G34588 | 1.46 |
| AT5G40981 | 1.43 |
| AT3G03341 | 1.40 |
| AT2G30766 | 1.39 |
| AT1G02850 | 1.38 |
| AT1G09500 | 1.38 |
| AT3G28210 | 1.38 |
| AT4G14690 | 1.38 |
| AT3G24982 | 1.38 |
| AT5G54165 | 1.35 |
| AT2G32210 | 1.34 |
| AT2G43820 | 1.31 |
| AT1G21525 | 1.31 |
| AT2G36750 | 1.30 |
| AT1G33960 | 1.30 |
| AT4G09153 | 1.30 |
| AT1G04684 | 1.30 |
| AT5G54610 | 1.29 |
| AT3G22600 | 1.28 |
| AT2G16367 | 1.27 |
| AT5G39520 | 1.25 |
| AT3G50480 | 1.24 |
| AT1G63840 | 1.24 |
| AT5G61820 | 1.24 |
| AT1G54100 | 1.24 |
| AT2G06822 | 1.24 |
| AT1G72680 | 1.23 |
| AT5G16980 | 1.23 |
| AT5G63065 | 1.23 |
| AT5G01870 | 1.23 |
| AT1G05560 | 1.23 |
| AT5G56300 | 1.22 |

| | |
|---|---|
| AT1G17830 | 1.91 |
| AT3G28740 | 1.89 |
| AT1G32960 | 1.87 |
| AT4G01895 | 1.85 |
| **AT2G37770** | 1.85 |
| AT4G01430 | 1.84 |
| **AT2G36750** | 1.84 |
| **AT2G29490** | 1.84 |
| AT3G46660 | 1.80 |
| **AT4G39670** | 1.79 |
| **AT2G43820** | 1.77 |
| AT1G15670 | 1.75 |
| AT5G22860 | 1.75 |
| AT4G23680 | 1.73 |
| AT2G16720 | 1.72 |
| AT3G46230 | 1.70 |
| **AT1G09500** | 1.69 |
| **AT3G28007** | 1.69 |
| AT5G48540 | 1.68 |
| AT2G29480 | 1.68 |
| AT5G53820 | 1.68 |
| AT3G11340 | 1.67 |
| AT1G62045 | 1.67 |
| AT3G21560 | 1.66 |
| **AT5G16980** | 1.66 |
| **AT1G29640** | 1.65 |
| AT4G12490 | 1.65 |
| AT2G45570 | 1.63 |
| **AT1G02850** | 1.63 |
| AT1G21390 | 1.62 |
| AT4G12500 | 1.62 |
| AT3G43270 | 1.61 |
| AT1G80160 | 1.60 |

| | | | |
|---|---|---|---|
| AT5G13330 | 1.21 | AT3G60970 | 1.60 |
| AT5G39610 | 1.21 | AT5G53710 | 1.59 |
| AT2G31271 | 1.21 | AT1G16515 | 1.57 |
| AT2G37760 | 1.21 | AT3G57010 | 1.56 |
| AT2G18660 | 1.20 | AT1G05560 | 1.55 |
| AT5G41109 | 1.19 | AT1G29195 | 1.55 |
| AT4G14630 | 1.19 | AT1G09510 | 1.55 |
| AT5G10760 | 1.19 | **AT3G17520** | 1.55 |
| AT5G35603 | 1.18 | AT5G19880 | 1.54 |
| AT1G10140 | 1.18 | **AT1G60730** | 1.53 |
| AT3G10912 | 1.16 | AT3G27025 | 1.52 |
| AT3G51860 | 1.15 | AT3G10910 | 1.52 |
| AT4G38620 | 1.15 | AT4G13395 | 1.52 |
| AT5G42460 | 1.14 | **AT5G59320** | 1.51 |
| AT4G39670 | 1.13 | **AT1G68620** | 1.51 |
| AT5G30545 | 1.13 | AT3G49780 | 1.51 |
| AT2G15310 | 1.13 | AT2G34070 | 1.48 |
| AT4G34135 | 1.13 | AT1G65690 | 1.48 |
| AT4G08967 | 1.13 | AT1G75040 | 1.48 |
| AT1G66390 | 1.12 | AT5G59220 | 1.47 |
| AT1G21520 | 1.12 | AT2G35980 | 1.46 |
| AT5G52810 | 1.12 | AT4G01870 | 1.46 |
| AT4G15248 | 1.12 | AT5G39050 | 1.45 |
| AT1G60730 | 1.11 | **AT5G61820** | 1.45 |
| AT4G37990 | 1.11 | AT4G34590 | 1.45 |
| AT5G15500 | 1.11 | AT5G07440 | 1.43 |
| AT1G43160 | 1.09 | AT2G45210 | 1.43 |
| AT3G22231 | 1.08 | AT5G13750 | 1.42 |
| AT3G58676 | 1.07 | **AT3G22060** | 1.42 |
| AT1G67360 | 1.07 | **AT1G21525** | 1.42 |
| AT1G29640 | 1.06 | AT1G51090 | 1.42 |
| AT4G30064 | 1.05 | AT1G21550 | 1.41 |
| AT3G47480 | 1.05 | **AT1G66390** | 1.41 |

| | | | |
|---|---|---|---|
| AT4G11250 | 1.04 | AT1G57590 | 1.40 |
| AT3G60647 | 1.04 | AT4G25810 | 1.40 |
| AT1G68620 | 1.04 | AT2G29470 | 1.40 |
| AT2G29460 | 1.04 | **AT5G39520** | 1.39 |
| AT3G22060 | 1.04 | AT4G28040 | 1.39 |
| AT2G17500 | 1.03 | AT2G05940 | 1.39 |
| AT1G77120 | 1.03 | **AT1G72680** | 1.38 |
| AT1G75900 | 1.02 | AT2G34810 | 1.38 |
| AT3G28007 | 1.02 | AT2G04515 | 1.38 |
| AT5G01300 | 1.01 | **AT3G44300** | 1.38 |
| AT3G42726 | 1.01 | **AT4G37990** | 1.37 |
| AT3G15536 | 1.01 | AT1G01720 | 1.37 |
| AT1G24580 | 1.01 | **AT5G54610** | 1.37 |
| AT5G18202 | 1.00 | AT4G02380 | 1.37 |
| AT2G38530 | 1.00 | AT2G39350 | 1.37 |
| AT1G12423 | 1.00 | **AT5G56300** | 1.36 |
| AT5G42500 | -1.00 | AT3G48020 | 1.34 |
| AT5G11412 | -1.02 | AT1G70800 | 1.34 |
| AT3G30247 | -1.02 | AT3G10450 | 1.34 |
| AT3G49744 | -1.02 | **AT2G17500** | 1.34 |
| AT4G15660 | -1.03 | AT5G27420 | 1.34 |
| AT2G30032 | -1.03 | AT5G13490 | 1.34 |
| AT5G34581 | -1.04 | AT4G20830 | 1.33 |
| AT4G36570 | -1.06 | AT2G14620 | 1.33 |
| AT5G47229 | -1.06 | AT3G48201 | 1.33 |
| AT1G75388 | -1.08 | AT2G39518 | 1.33 |
| AT2G06906 | -1.09 | AT5G39550 | 1.32 |
| AT4G39250 | -1.12 | AT5G24110 | 1.32 |
| AT5G31807 | -1.16 | **AT2G37760** | 1.31 |
| AT2G38544 | -1.20 | AT5G44420 | 1.31 |
| AT4G06496 | -1.22 | AT1G24686 | 1.31 |
| AT4G30872 | -1.23 | AT4G24000 | 1.30 |
| AT3G29480 | -1.23 | AT1G62570 | 1.30 |

| | |
|---|---|
| AT3G30778 | -1.28 |
| AT3G42083 | -1.30 |
| AT2G42870 | -1.33 |
| AT1G62978 | -1.34 |
| AT1G17285 | -1.35 |
| AT5G28886 | -1.36 |
| AT1G54730 | -1.36 |
| AT1G44941 | -1.64 |
| AT4G15061 | -1.69 |
| AT4G06627 | -1.95 |

| | |
|---|---|
| AT3G54420 | 1.30 |
| AT4G20860 | 1.30 |
| **AT5G39610** | 1.30 |
| **AT2G47770** | 1.29 |
| AT2G22470 | 1.29 |
| AT3G46080 | 1.29 |
| AT4G37370 | 1.29 |
| AT2G23150 | 1.29 |
| AT3G25221 | 1.28 |
| AT2G47950 | 1.27 |
| AT2G35070 | 1.27 |
| **AT2G15310** | 1.27 |
| **AT4G34588** | 1.27 |
| AT2G37710 | 1.27 |
| AT1G69870 | 1.26 |
| **AT3G50480** | 1.26 |
| **AT2G29350** | 1.25 |
| AT5G56630 | 1.25 |
| AT2G35612 | 1.25 |
| **AT1G05340** | 1.25 |
| AT3G14620 | 1.25 |
| AT2G15490 | 1.25 |
| AT3G26690 | 1.24 |
| AT5G24090 | 1.24 |
| AT2G41190 | 1.23 |
| **AT3G24982** | 1.22 |
| AT5G24660 | 1.22 |
| AT5G10695 | 1.22 |
| AT1G68570 | 1.22 |
| **AT5G52810** | 1.21 |
| AT5G59820 | 1.21 |
| **AT3G28210** | 1.21 |
| AT1G79410 | 1.21 |

| | |
|---|---|
| **AT5G54165** | 1.21 |
| AT2G36800 | 1.21 |
| AT1G08120 | 1.20 |
| AT1G28660 | 1.20 |
| AT4G07943 | 1.20 |
| **AT1G24580** | 1.20 |
| AT5G58840 | 1.20 |
| AT4G23150 | 1.19 |
| AT3G22100 | 1.19 |
| AT5G57785 | 1.19 |
| AT1G02470 | 1.19 |
| AT4G12470 | 1.18 |
| AT2G37750 | 1.18 |
| AT4G13180 | 1.17 |
| AT3G28340 | 1.17 |
| AT4G23600 | 1.17 |
| **AT2G33380** | 1.17 |
| AT4G25640 | 1.16 |
| **AT1G67360** | 1.16 |
| AT4G06694 | 1.16 |
| AT5G03210 | 1.16 |
| AT1G17020 | 1.16 |
| AT2G32190 | 1.16 |
| **AT1G54100** | 1.15 |
| AT3G11480 | 1.15 |
| AT1G26770 | 1.15 |
| AT1G14130 | 1.14 |
| **AT2G38530** | 1.14 |
| AT5G57391 | 1.14 |
| AT3G55470 | 1.14 |
| AT5G17390 | 1.14 |
| AT3G28956 | 1.13 |
| AT1G70645 | 1.13 |

| | |
|---|---|
| AT5G26280 | 1.13 |
| AT5G24080 | 1.13 |
| AT4G36990 | 1.12 |
| AT2G35300 | 1.12 |
| AT3G14060 | 1.12 |
| **AT5G13330** | 1.12 |
| **AT5G06760** | 1.12 |
| AT5G16450 | 1.11 |
| AT5G13170 | 1.11 |
| AT1G76970 | 1.11 |
| AT5G17380 | 1.11 |
| AT5G67480 | 1.11 |
| AT2G33590 | 1.11 |
| **AT4G14630** | 1.11 |
| AT5G42053 | 1.11 |
| AT5G44990 | 1.11 |
| AT2G29420 | 1.11 |
| AT3G56710 | 1.10 |
| AT4G34710 | 1.10 |
| AT1G56650 | 1.10 |
| AT5G64120 | 1.09 |
| AT1G59590 | 1.09 |
| AT1G07985 | 1.09 |
| AT2G46662 | 1.09 |
| AT5G59845 | 1.08 |
| AT1G57560 | 1.08 |
| AT3G17110 | 1.08 |
| AT4G22530 | 1.08 |
| **AT1G10140** | 1.07 |
| AT5G16970 | 1.07 |
| AT1G22470 | 1.07 |
| AT2G11507 | 1.07 |
| AT2G26020 | 1.07 |

| | |
|---|---|
| AT3G08860 | 1.07 |
| AT2G34500 | 1.07 |
| AT4G07917 | 1.06 |
| AT1G59860 | 1.06 |
| AT3G12830 | 1.06 |
| AT5G17760 | 1.06 |
| AT4G31870 | 1.06 |
| AT4G39675 | 1.05 |
| AT4G15490 | 1.05 |
| AT1G68440 | 1.05 |
| AT1G02220 | 1.05 |
| AT5G67600 | 1.05 |
| AT4G39986 | 1.05 |
| AT1G68945 | 1.05 |
| AT5G22460 | 1.05 |
| AT5G27760 | 1.04 |
| AT4G33540 | 1.04 |
| AT1G75270 | 1.04 |
| AT5G35735 | 1.03 |
| AT2G36830 | 1.03 |
| AT3G09270 | 1.03 |
| AT5G49480 | 1.03 |
| AT2G40340 | 1.03 |
| AT5G48180 | 1.03 |
| AT3G49120 | 1.03 |
| AT5G42380 | 1.03 |
| AT1G71370 | 1.02 |
| AT5G13200 | 1.01 |
| AT5G12020 | 1.01 |
| AT4G08555 | 1.01 |
| AT3G28857 | 1.01 |
| AT1G32940 | 1.01 |
| AT4G33150 | 1.00 |

| | |
|---|---|
| AT2G24100 | 1.00 |
| AT3G28220 | -1.00 |
| AT3G29670 | -1.00 |
| AT3G29680 | -1.01 |
| AT5G03130 | -1.01 |
| AT2G15890 | -1.01 |
| AT1G21130 | -1.02 |
| AT3G49110 | -1.02 |
| AT4G39795 | -1.04 |
| AT2G11522 | -1.04 |
| AT3G43640 | -1.04 |
| AT5G32022 | -1.06 |
| AT1G32780 | -1.06 |
| AT5G22920 | -1.06 |
| AT1G74670 | -1.07 |
| AT2G32885 | -1.07 |
| AT3G06070 | -1.07 |
| AT5G48490 | -1.07 |
| AT4G01595 | -1.08 |
| AT2G37040 | -1.08 |
| AT1G27860 | -1.08 |
| AT3G53800 | -1.09 |
| AT5G06690 | -1.09 |
| AT5G26220 | -1.09 |
| AT5G25190 | -1.10 |
| AT3G58850 | -1.10 |
| AT3G16770 | -1.11 |
| AT2G27385 | -1.11 |
| AT1G80745 | -1.11 |
| AT4G27450 | -1.12 |
| AT1G61575 | -1.12 |
| **AT4G15660** | -1.12 |
| AT2G26750 | -1.13 |

| | |
|---|---|
| AT3G53260 | -1.13 |
| AT2G39705 | -1.13 |
| AT1G66100 | -1.14 |
| AT4G04840 | -1.14 |
| AT5G54585 | -1.15 |
| AT1G06360 | -1.16 |
| AT1G20190 | -1.17 |
| AT3G28216 | -1.19 |
| AT2G33830 | -1.19 |
| AT4G18970 | -1.19 |
| AT1G36130 | -1.20 |
| AT1G36550 | -1.21 |
| AT2G34825 | -1.21 |
| AT3G13404 | -1.22 |
| AT3G15450 | -1.22 |
| AT1G56600 | -1.23 |
| AT5G02021 | -1.25 |
| AT1G21120 | -1.26 |
| AT5G43695 | -1.26 |
| AT1G34405 | -1.30 |
| AT3G08770 | -1.30 |
| AT2G30424 | -1.32 |
| AT3G01960 | -1.32 |
| AT3G61060 | -1.34 |
| AT4G22520 | -1.35 |
| AT3G62950 | -1.35 |
| **AT1G12423** | -1.36 |
| AT2G22980 | -1.36 |
| AT2G40610 | -1.37 |
| AT5G39860 | -1.38 |
| AT4G36410 | -1.39 |
| AT3G33081 | -1.39 |
| AT2G18042 | -1.40 |

| | |
|---|---|
| AT2G20605 | -1.40 |
| **AT2G42870** | -1.44 |
| AT4G29290 | -1.44 |
| AT1G75250 | -1.57 |
| AT5G36910 | -1.62 |
| AT3G22540 | -1.66 |
| AT5G02550 | -1.77 |
| AT1G06080 | -1.77 |
| AT1G67400 | -1.82 |
| AT3G05727 | -1.94 |
