# Supplementary material for: Poly(ADP-Ribose)Polymerase Activity Controls Plant Growth by Promoting Leaf Cell Number
Source: PLoS One. 2014 Feb 28;9(2):e90322. doi: 10.1371/journal.pone.0090322 (PMC3938684; doi:10.1371/journal.pone.0090322)
Supplement: Table S2 — Complete list of detected primary metabolites. For the GC-MS analysis samples were taken in parallel to samples for the physiological measurements for both conditions (+/− 3MB) at day1, day2, day4 and day7. The experiment was repeated three times independently with five replicates each (n = 15). Relative abundance of metabolite in the 3MB treated sample compared to the control samples are shown. (PDF) [file pone.0090322.s006.pdf]

Table 6.1 Primary Metabolites

Amino acids and derivates	Day1	Day2	Day4	Day7
Aspartate	**0.78**	0.95	**0.35**	1.10
Asparagine	**3.33**	**1.38**	0.99	0.42
Threonine	**0.83**	1.03	1.26	1.07
Isoleucine	0.97	1.64	0.77	1.28
Methionine	1.08	1.22	0.87	0.94
Pyroglutamat	**0.72**	2.16	1.09	**1.43**
Glutamat	1.08	1.27	1.11	1.09
Glutamine	0.78	1.07	0.92	1.07
Ornithine	1.35	**1.96**	0.45	1.11
Proline	0.91	0.82	0.96	**0.32**
Arginine	1.00	2.38	0.98	0.74
Spermidine	0.81	**0.58**	0.95	0.97
Tryptophan	1.02	0.88	0.98	0.68
Tyramine	0.84	0.85	0.91	**1.33**
Tyrosine	**2.55**	1.48	1.07	0.64
Serine	0.78	**1.42**	1.39	1.06
Homoserine	0.54	0.74	0.66	1.11
Glycine	1.03	0.96	**1.73**	0.86
Cysteine	1.30	1.04	1.05	0.79
beta_alanine	**3.38**	0.89	**0.50**	**0.22**
Alanine	0.96	**1.23**	1.04	0.95
Histidine	1.20	0.88	0.66	**0.43**
Leucine	**0.83**	1.37	1.47	**1.54**
Valine	1.06	1.03	1.11	1.11
**Organic acids**				
Pyruvic acid	**0.69**	1.00	0.67	1.12
Citric acid	1.05	0.82	0.67	1.19
Fumaric acid	**0.74**	0.91	0.87	**1.56**
Malic acid	**1.59**	0.75	1.56	0.94
Succinic acid	1.43	0.68	1.45	0.66
2-Oxoglutarat	**0.60**	1.14	1.20	1.10
4-Aminobutyrat	0.90	0.91	**0.57**	**0.60**
Ribonic acid	0.86	**0.35**	**0.33**	**0.42**
Shikimic acid	**0.67**	0.84	0.73	**1.35**
Gluconic acid	1.09	1.42	1.23	0.88
Glyceric acid	1.20	1.11	1.04	1.14
Glyceric acid 3-P	0.69	0.97	1.35	1.17
**Carbohydrates**				
Arabinose	**0.89**	**1.15**	1.11	1.20
Arabitol	1.03	1.38	0.69	1.43
Erythritol	**0.50**	**2.00**	0.77	**1.55**
Fructose	1.07	0.80	0.73	**0.21**
Fructose-6-P	**3.71**	0.67	0.87	0.92
3,6-anhydro-Galactose	1.33	0.90	**0.62**	0.99
Isomaltose	1.18	0.81	0.93	**0.80**
Maltose	0.89	0.98	1.09	1.11

Raffinose	**0.69**	**2.01**	**2.37**	1.27
Sorbose	1.16	1.38	0.53	1.00
Sucrose	**0.92**	0.89	1.15	1.20
Trehalose	1.20	1.07	1.19	1.31
1,6-Anhydro-Glucose	0.82	1.02	0.86	1.19
**Fatty acids**				
Glycerol	**0.23**	**0.58**	**0.57**	1.33
Docosanoic acid	**0.50**	0.82	0.61	0.41
Hexacosanoic acid	**0.55**	0.97	1.32	1.01
Octadecanoic acid	**0.62**	0.87	1.18	1.41
Palmitic acid	1.14	0.99	0.99	1.08
Tetracosanoic acid	0.93	1.09	0.80	0.56
**Others**				
Myoinositol	1.20	1.07	**0.65**	0.96
Nicotinate	2.62	**0.33**	**0.35**	**0.34**
4-Hydroxycinnamaic acid	0.94	1.69	1.13	1.34
Indole-3-acetonitrile	**1.44**	0.79	1.19	1.29
Glycerol-3P	**2.88**	0.92	1.30	0.91
AMP	0.86	1.12	1.22	0.95
Adenine	0.90	1.22	0.68	1.19
Adenosine-5-P	0.86	1.12	1.22	0.95
Putrescine	1.34	0.92	**1.45**	0.92
